# Supplementary material for: The Heptaprenyl Diphosphate Synthase (Coq1) Is the Target of a Lipophilic Bisphosphonate That Protects Mice against Toxoplasma gondii Infection
Source: mBio. 2022 Sep 21;13(5):e01966-22. doi: 10.1128/mbio.01966-22 (PMC9600589; doi:10.1128/mbio.01966-22)
Supplement: TABLE S1 [file mbio.01966-22-s0006.pdf]

Supplementary Table S1: Sequences used for the phylogenetic analysis presented in Figure 1.

Organism_Enzyme	GenBank/EuPathDB ID	Reference
*Arabidopsis thaliana*_SPPS	NP_177972.2	(1)
*Saccharomyces cerevisiae*_HexPPS	P18900.1	(2)
*Trypanosoma cruzi*_SPPS	EAN82722.1/ Tc00.1047053427091.10	(3)
*Leishmania major*_SPPS	XP_001682016.1/LmjF.15.1020	Predicted
*Plasmodium vivax*_OPPS	VUZ93874.1/PVX_003575	Predicted
*Plasmodium falciparum*_OPPS	XP_001349541.1/PFB0130w	(4)
*Toxoplasma gondii*_LONG	XP_018636584.1/TGME49_069430	This work
*Neospora caninum*_PPS	XP_003883948.1/NCLIV_036980	Predicted
*Oryza sativa*_SPPS	Q653T6.1	(5)
*Babesia bovis*_PPS	EDO07087.1	Predicted
*Haemophilus influenzae*_HepPPS	CBY85984.1	Predicted
Bacillus subtilis HepPPS	ARW31988.1	(6)
Listeria monocytogenes HepPPS	QGK56962.1	Predicted
Aquifex aeolicus OPPS	NP_213606.1	Predicted
Aspergillus fumigatus HexPPS	EDP53123.1	Predicted
Mus musculus SPPS	BAE48219.1	(7)
Equus caballus DPPS	XP023487828.1	Predicted
Homo sapiens DPPS	BAE48216.1	(7)
Hammondia hammondi PPS	HHA_269430/HHA_269430	Predicted
Cyclospora cayetanensis PPS	LOC34620908	Predicted
Sarcocystis neurona PPS	SN3_03700020	Predicted

References

1. Jun L, Saiki R, Tatsumi K, Nakagawa T, Kawamukai M. 2004. Identification and subcellular localization of two solanesyl diphosphate synthases from Arabidopsis thaliana. Plant Cell Physiol 45:1882-8.
2. Ashby MN, Edwards PA. 1990. Elucidation of the deficiency in two yeast coenzyme Q mutants. Characterization of the structural gene encoding hexaprenyl pyrophosphate synthetase. J Biol Chem 265:13157-64.
3. Ferella M, Montalvetti A, Rohloff P, Miranda K, Fang J, Reina S, Kawamukai M, Bua J, Nilsson D, Pravia C, Katzin A, Cassera MB, Aslund L, Andersson B, Docampo R, Bontempi EJ. 2006. A solanesyl-diphosphate synthase localizes in glycosomes of Trypanosoma cruzi. J Biol Chem 281:39339-48.
4. Tonhosolo R, D'Alexandri FL, Genta FA, Wunderlich G, Gozzo FC, Eberlin MN, Peres VJ, Kimura EA, Katzin AM. 2005. Identification, molecular cloning and functional characterization of an octaprenyl pyrophosphate synthase in intra-erythrocytic stages of Plasmodium falciparum. Biochem J 392:117-26.

5. Ohara K, Sasaki K, Yazaki K. 2010. Two solanesyl diphosphate synthases with different subcellular localizations and their respective physiological roles in Oryza sativa. J Exp Bot 61:2683-92.
6. Zhang YW, Koyama T, Marecak DM, Prestwich GD, Maki Y, Ogura K. 1998. Two subunits of heptaprenyl diphosphate synthase of Bacillus subtilis form a catalytically active complex. Biochemistry 37:13411-20.
7. Saiki R, Nagata A, Kainou T, Matsuda H, Kawamukai M. 2005. Characterization of solanesyl and decaprenyl diphosphate synthases in mice and humans. FEBS J 272:5606-22.
