# Supplementary material for: The Heptaprenyl Diphosphate Synthase (Coq1) Is the Target of a Lipophilic Bisphosphonate That Protects Mice against Toxoplasma gondii Infection
Source: mBio. 2022 Sep 21;13(5):e01966-22. doi: 10.1128/mbio.01966-22 (PMC9600589; doi:10.1128/mbio.01966-22)

Supplementary Table S2: physicochemical properties of the TgCoq1 N terminal domain

A

$_{32}$FFSSLSCVSVPPPLCAVS$_{49}$		
**Physico-chemical properties**	**Polar residues + GLY**	**Nonpolar residues**
Hydrophobicity <H>	Polar residues + GLY (n / %)	Nonpolar residues (n / %)
0.888	5 / 27.78	13 / 72.22
Hydrophobic moment <μH>	Uncharged residues + GLY	Aromatic residues
0.073	SER 5, GLY 0	PHE 2
Net charge z	Charged residues	Special residues
0		CYS 2, PRO 3
**Hydrophobic face:** C V F P L A		

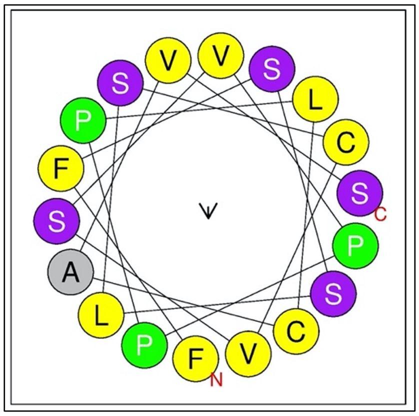

B

$_{68}$VRGEDRRRLSGRRRQVEK$_{85}$		
**Physico-chemical properties**	**Polar residues + GLY**	**Nonpolar residues**
Hydrophobicity <H>	Polar residues + GLY (n / %)	Nonpolar residues (n / %)
-0.346	15 / 83.33	3 / 16.67
Hydrophobic moment <μH>	Uncharged residues + GLY	Aromatic residues
0.212	GLN 1, SER 1, GLY 2	
Net charge z	Charged residues	Special residues
5	LYS 1, ARG 7, GLU 2, ASP 1	CYS 0, PRO 0
**Hydrophobic face:** none		

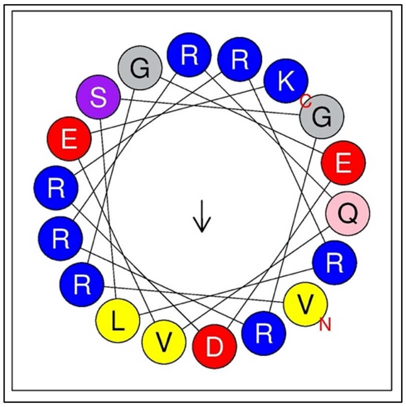

C

$_{185}$EGSEDEGTTSEQPSGGRD$_{202}$		
**Physico-chemical properties**	**Polar residues + GLY**	**Nonpolar residues**
Hydrophobicity <H>	Polar residues + GLY (n /%)	Nonpolar residues (n /%)
-0.234	17 / 94.44	1 / 5.56
Hydrophobic moment <μH>	Uncharged residues + GLY	Aromatic residues
0.082	GLN 1, SER 3, THR 2, GLY 4	
Net charge z	Charged residues	Special residues
-5	ARG 1, GLU 4, ASP 2,	CYS 0, PRO 1
**Hydrophobic face:** none		

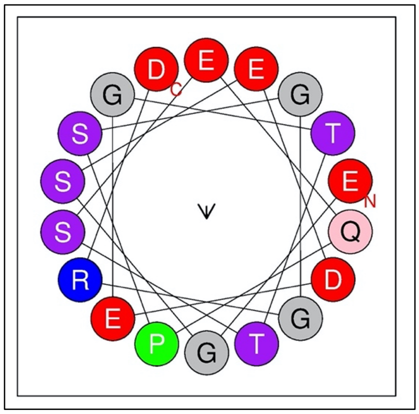

Supplement: TABLE S2 [file mbio.01966-22-s0007.pdf]
