# Supplementary material for: The Heptaprenyl Diphosphate Synthase (Coq1) Is the Target of a Lipophilic Bisphosphonate That Protects Mice against Toxoplasma gondii Infection
Source: mBio. 2022 Sep 21;13(5):e01966-22. doi: 10.1128/mbio.01966-22 (PMC9600589; doi:10.1128/mbio.01966-22)
Supplement: FIG S2 [file mbio.01966-22-s0002.pdf]

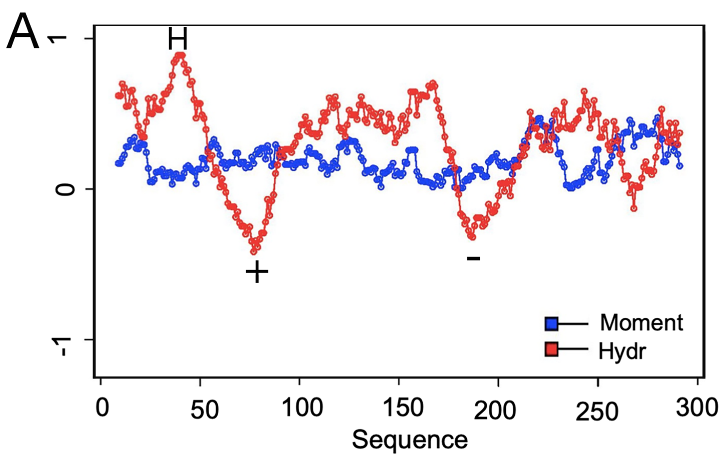

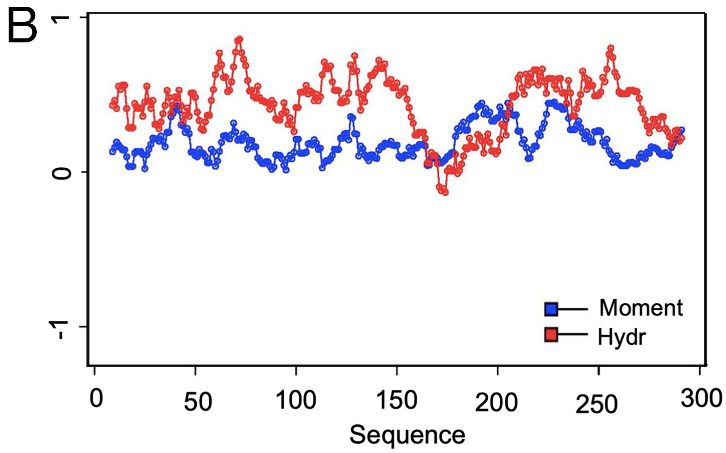

Supplementary Figure S2. A, Hydrophobicity <**H**> (red) and hydrophobic moment <**μH**> (blue) as a function of sequence position for the first 300 residues in TgCoq1; H=hydrophobic, the "+" and "-" values indicate the Arg/Lys and Asp/Glu-rich regions. **B**, Hydrophobicity <**H**> (red) and hydrophobic moment <**μH**> (blue) as a function of sequence position for the first 300 residues in TgFPPS.
