# Supplementary material for: The Heptaprenyl Diphosphate Synthase (Coq1) Is the Target of a Lipophilic Bisphosphonate That Protects Mice against Toxoplasma gondii Infection
Source: mBio. 2022 Sep 21;13(5):e01966-22. doi: 10.1128/mbio.01966-22 (PMC9600589; doi:10.1128/mbio.01966-22)
Supplement: FIG S3 [file mbio.01966-22-s0004.pdf]

A

Trial	UQ_7 (µg/1x10^{10}parasites)
1	4.956
2	3.389
Average	4.173

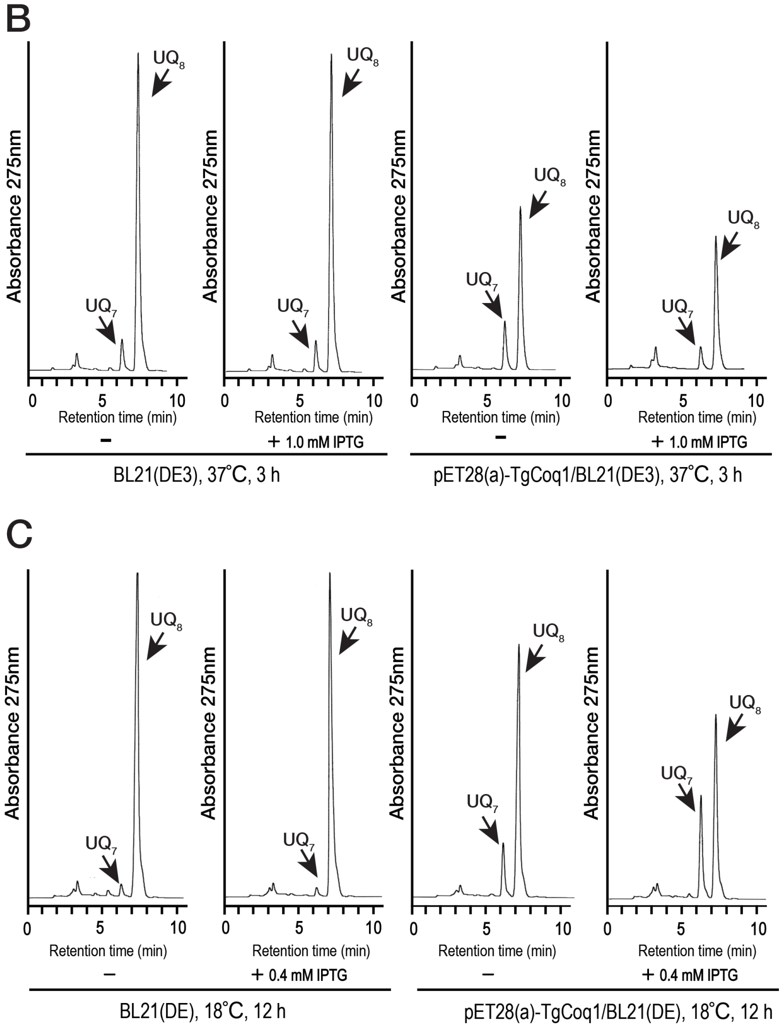

Supplementary Figure S3. (A) Amount of Ubiquinone Q_7 present in RH wild-type parasites. UQ was measured using HPLC, and the amount of UQ_7 present was calculated based on an internal UQ_{10} standard. (B-C) Expression of the entire length of the *TgCoq1* gene cloned in the pET28(a) plasmid under the control of the T7 lac promoter. UQ was extracted from *E. coli* BL21(DE3) harboring pET28(a)-TgCoq1, in which TgCoq1 was induced by addition of isopropyl β-D-thiogalactopyranoside (IPTG) (1.0 mM) at 37°C for 3 hours (B) or 0.4 mM at 18°C for 12 hours (C). They were first separated by TLC and further analyzed by HPLC. These results showed that *E. coli* BL21(DE3) expressing TgCoq1 induced by 0.4 mM IPTG at 18°C for 12 hours clearly produced UQ_7 but did not so much by 1.0 mM IPTG at 37 °C for 3 hours.
