# Supplementary material for: The Heptaprenyl Diphosphate Synthase (Coq1) Is the Target of a Lipophilic Bisphosphonate That Protects Mice against Toxoplasma gondii Infection
Source: mBio. 2022 Sep 21;13(5):e01966-22. doi: 10.1128/mbio.01966-22 (PMC9600589; doi:10.1128/mbio.01966-22)
Supplement: FIG S4 [file mbio.01966-22-s0003.pdf]

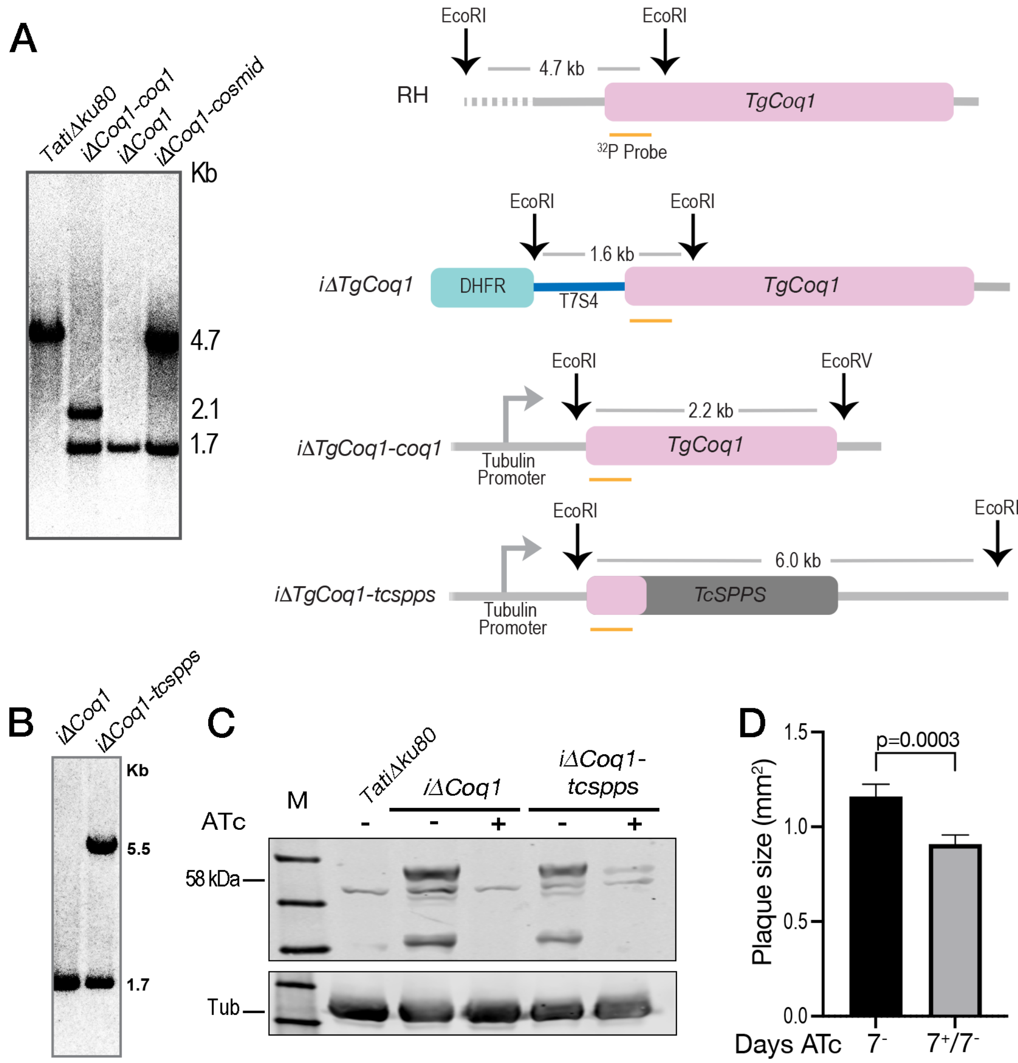

Supplemental Figure S4. A, Southern blot analysis of genomic DNA isolated from the *iΔCoq1*, *iΔCoq1*-Coq1, and *iΔCoq1*-cosmid complementation mutants. The model depicts the constructs of each of the cell lines, and the location of the enzymes cut sites for the southern blot analysis. **B,** Southern blot analysis of the *iΔCoq1-tcspps* complementation mutant. **C,** Western blot analysis with HA antibody showing the HA expression in the *iΔCoq1*-ATc (58 kDA) and the loss of HA signal in the *iΔCoq1* with 3 days ATc. The western also shows the HA signal in the *iΔCoq1-tcspps* (56 kDA). **D,** Plaque size quantification of growth for the *iΔCoq1* cell line grown with ATc for 7 days and then 7 days without ATc ($7^+/7^-$) compared to plaques formed by the control mutant (-ATc) for 7 days (7^-). Average from 3 biological replicates, Student's T-test statistical analysis.
