# Supplementary material for: The Heptaprenyl Diphosphate Synthase (Coq1) Is the Target of a Lipophilic Bisphosphonate That Protects Mice against Toxoplasma gondii Infection
Source: mBio. 2022 Sep 21;13(5):e01966-22. doi: 10.1128/mbio.01966-22 (PMC9600589; doi:10.1128/mbio.01966-22)
Supplement: FIG S5 [file mbio.01966-22-s0005.pdf]

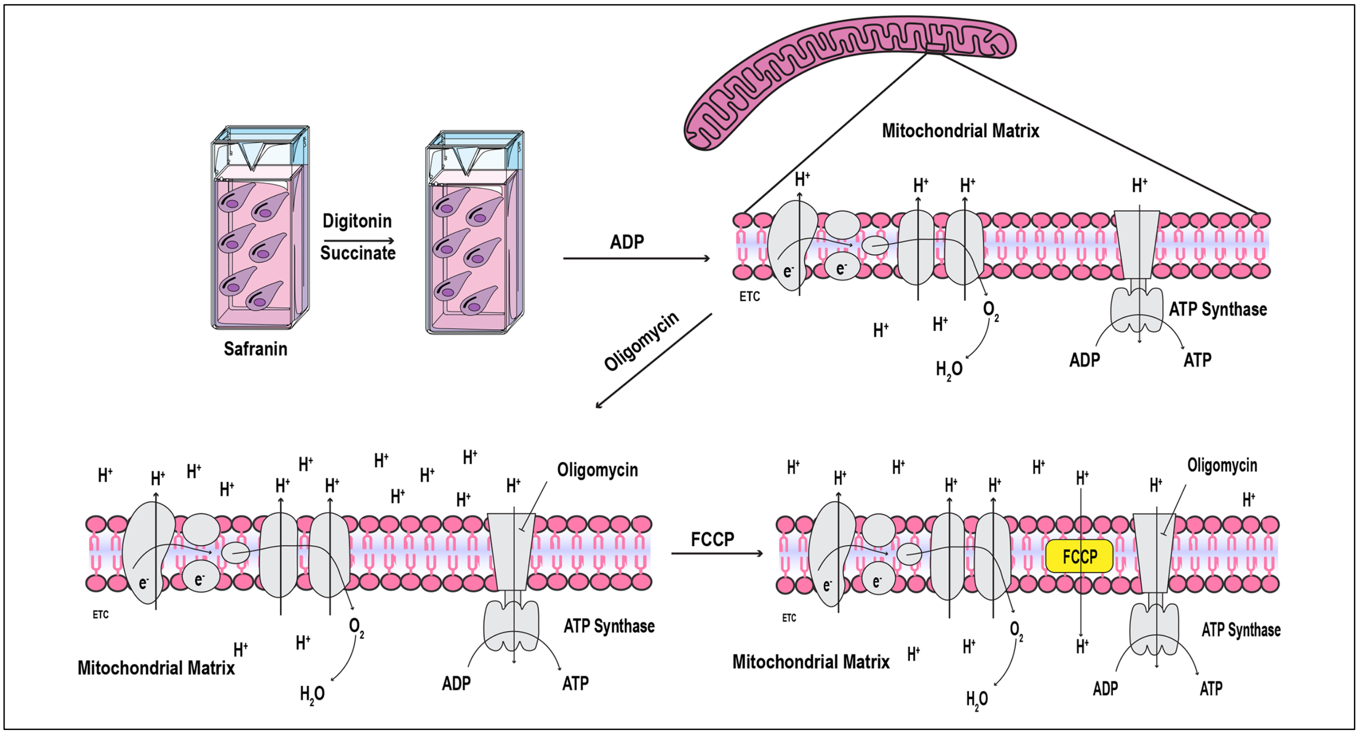

Supplementary Figure S5. Model of mitochondrial membrane potential experiments. Digitonin (Dig) addition permeabilizes the plasma membrane for the mitochondrial substrate, succinate. Adenosine diphosphate (ADP) results in synthesis of ATP and use of the proton gradient. Oligomycin (Oligo) inhibits the ATP synthase and allows the membrane potential to recover. Carbonyl cyanide-4-(trifluoromethoxy) phenylhydrazone (FCCP) is a proton ionophore and collapses the H^+ gradient.
