# Supplementary material for: The Heptaprenyl Diphosphate Synthase (Coq1) Is the Target of a Lipophilic Bisphosphonate That Protects Mice against Toxoplasma gondii Infection
Source: mBio. 2022 Sep 21;13(5):e01966-22. doi: 10.1128/mbio.01966-22 (PMC9600589; doi:10.1128/mbio.01966-22)
Supplement: TABLE S3 [file mbio.01966-22-s0008.pdf]

Supplementary Table S3: Cytotoxicity of compounds on hTERT cells at 4X and 10X their EC_{50}. Values are means ± s.d. of n = 2.

Compound (μM)	% Inhibition
**BPH-1218 (4)**	4.30 ± 2.31
**BPH-1218 (10)**	10.28 ± 1.07
**BPH-1217 (28.4)**	8.01 ± 1.82
**BPH-1217 (71)**	6.19 ± 9.55
**BPH-1219 (46)**	3.20 ± 2.89
**BPH-1219 (115)**	8.01 ± 1.82
**BPH-1236 (2.36)**	4.13 ± 1.20
**BPH-1236 (5.9)**	9.10 ± 2.38
**BPH-1238 (2.72)**	9.77 ± 1.18
**BPH-1238 (6.8)**	13.37 ± 6.08
**AV (0.12)**	2.33 ± 0.99
**AV (0.32)**	2.91 ± 4.90
**JAG-21 (0.48)**	6.40 ± 5.50
**JAG-21 (1.2)**	5.75 ± 4.98
