# Supplementary material for: The Heptaprenyl Diphosphate Synthase (Coq1) Is the Target of a Lipophilic Bisphosphonate That Protects Mice against Toxoplasma gondii Infection
Source: mBio. 2022 Sep 21;13(5):e01966-22. doi: 10.1128/mbio.01966-22 (PMC9600589; doi:10.1128/mbio.01966-22)
Supplement: TABLE S5 [file mbio.01966-22-s0010.pdf]

Supplementary Table S5: Primers used in this work

Primer	Primer Use	Sequence
1	In situ tagging	tacttccaatccaatttaatgcAGACAACAGCGCAGTCCAAGTCTTG
2	In situ tagging	tcctccacttccaattttagcCCCAGACCGCCGCTGGAGAGTGGCC
3	Promoter Insertion 5' flanking sequence fragment	CATATGAAATTAGACAGAAGTGCCGAGAAG
4	Promoter Insertion 5' flanking sequence fragment	CATATGTTCGCCGACAGACACAAGAGAGATC
5	Promoter Insertion 5' coding sequence fragment	AGATCTATGACGCTCGTTACTCGACACC
6	Promoter Insertion 5' coding sequence fragment	CCTAGGGATTCAGAAACGAATTACAGAG
7	Full-length cDNA F	AGATCTATGACGCTCGTTACTCGACACC
8	Full-length cDNA R	CCTAGGGATTCAGAAACGAATTACAGAG
