# Supplementary material for: The Heptaprenyl Diphosphate Synthase (Coq1) Is the Target of a Lipophilic Bisphosphonate That Protects Mice against Toxoplasma gondii Infection
Source: mBio. 2022 Sep 21;13(5):e01966-22. doi: 10.1128/mbio.01966-22 (PMC9600589; doi:10.1128/mbio.01966-22)
Supplement: TABLE S4 [file mbio.01966-22-s0009.pdf]

Supplementary Table S4: Compounds for CoQ6 rescue experiment from Fig. 7D.

Compound	Structure	Compound	Structure
BPH-1218	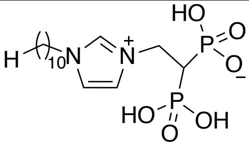	BPH-754	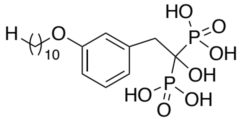
BPH-1327	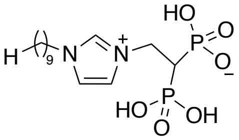	Risedronate	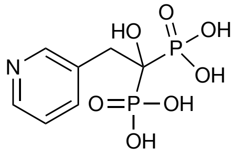
BPH-1236	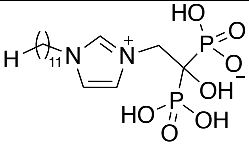	C7S	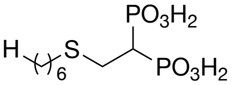
JAG-21	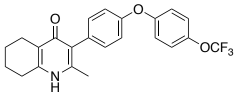	CE-22	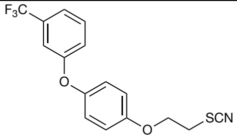
BPH-1238	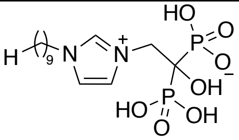	MNC-98	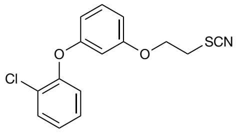
Atovaquone	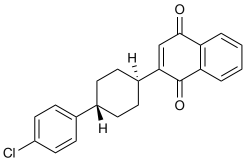	CE-29	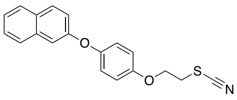
Pyrimethamine	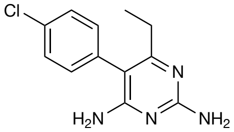	MNCA181	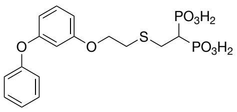
BPH-1217	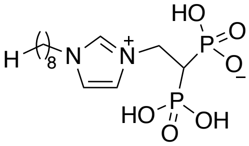	CE-109	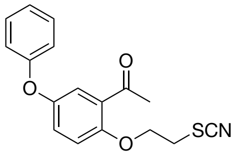
BPH-1219	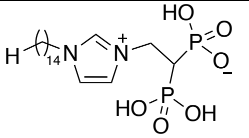	CE-91	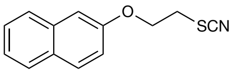
