# Supplementary material for: The Heptaprenyl Diphosphate Synthase (Coq1) Is the Target of a Lipophilic Bisphosphonate That Protects Mice against Toxoplasma gondii Infection
Source: mBio. 2022 Sep 21;13(5):e01966-22. doi: 10.1128/mbio.01966-22 (PMC9600589; doi:10.1128/mbio.01966-22)
Supplement: FIG S1 [file mbio.01966-22-s0001.pdf]

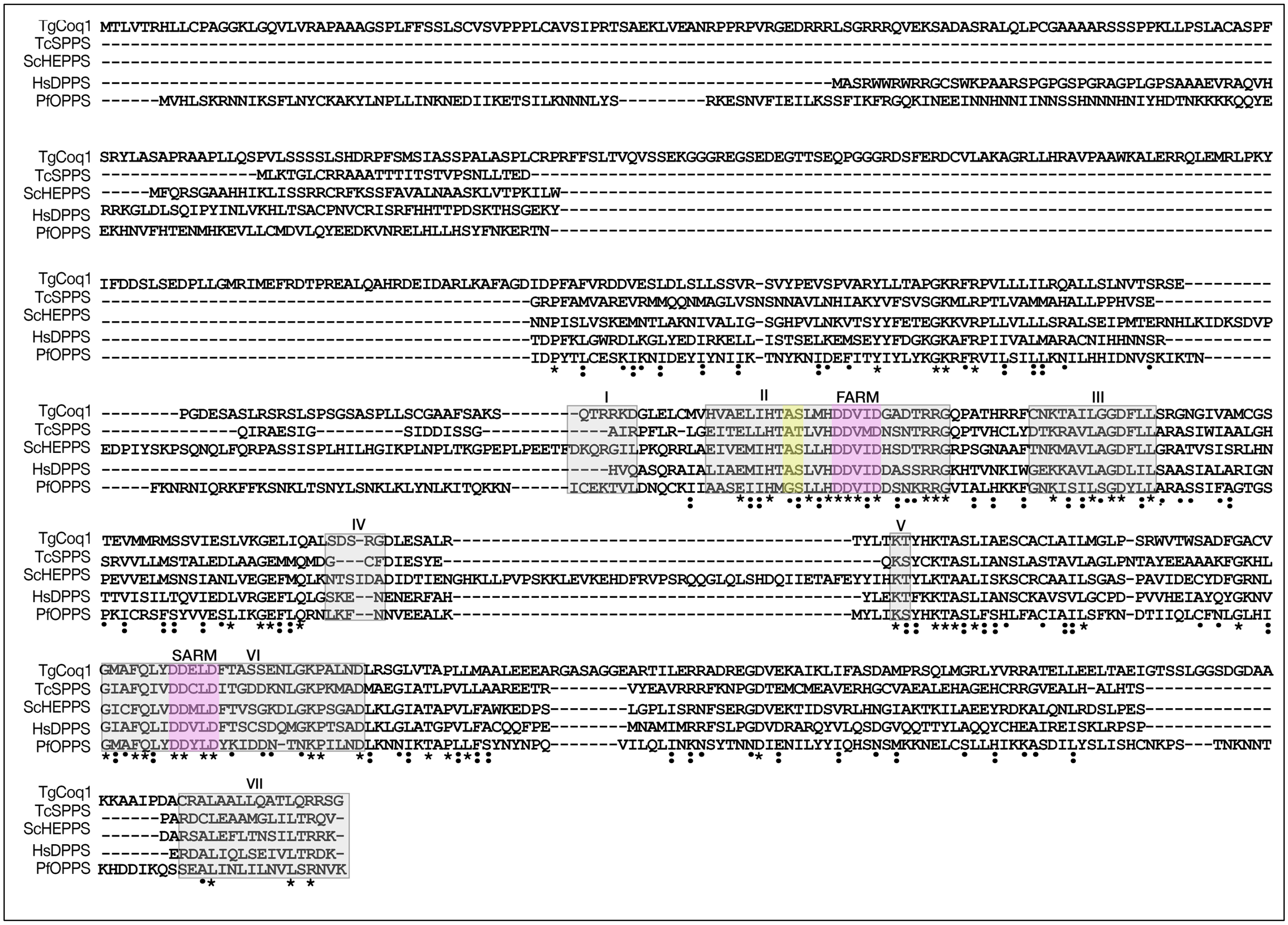

Supplementary Figure S1. Clustal sequence alignment of the protein sequences from *T. gondii* Coq1 (TgCoq1), a solanesyl diphosphate synthase from *T. cruzi* (TcSPPS), an octaprenyl diphosphate synthase from *P. falciparum* (PfOPPS), a Hexaprenyl diphosphate synthase from *S. cerevisiae* (SceHexPPS) and the human decaprenyl diphosphate synthase (HsDPPS). The first aspartic rich domain (FARM) and the second aspartic rich domain (SARM) are highlighted in pink and are conserved amongst the sequences. The 4th and 5th positions prior to the FARM are highlighted in yellow. *: highlights identical amino acids between the sequences. The other symbols indicate partial conservation between sequences.
